# Examining and Promoting Sleep Health in the Undergraduate Classroom: A Mixed-Methods Approach

**DOI:** 10.3390/ijerph182312297

**Published:** 2021-11-23

**Authors:** Natalie D. Dautovich, Ashley R. MacPherson, Sarah M. Ghose, Claire M. Williams, Morgan P. Reid, Sahar M. Sabet, Pablo Soto, Shawn C. T. Jones, Joseph M. Dzierzewski

**Affiliations:** Department of Psychology, Virginia Commonwealth University, P.O. Box 842018, Richmond, VA 23284, USA; macphersona@vcu.edu (A.R.M.); ghosesm@vcu.edu (S.M.G.); williamscm2@vcu.edu (C.M.W.); reidmp@vcu.edu (M.P.R.); sabetsm@vcu.edu (S.M.S.); sotop2@vcu.edu (P.S.); scjones4@vcu.edu (S.C.T.J.); dzierzewski@vcu.edu (J.M.D.)

**Keywords:** sleep health, self-determination, college students, undergraduates, RU SATED, insomnia

## Abstract

**Objective:** Although college students are at heightened risk for sleep disturbances, healthy sleep is associated with positive physical, cognitive, psychological, and academic benefits for this group. The goals of the current study were to (1) describe sleep health in an undergraduate college sample and (2) examine the role of a class activity using self-determination theory to promote better sleep health in this group. **Methods:** A cohort study was conducted using data drawn from class activities conducted in two undergraduate Introduction to Psychology courses. Students were undergraduates at a mid-Atlantic public university in the United States. Total sample size was *N* = 224 (intervention class [*n* = 98], and the control class [*n* = 127]). Both the intervention and control classes completed the RU SATED sleep health questionnaire at the beginning and the end of the semester. The intervention class also completed a self-determination activity focused on sleep health mid-semester. Both the RU SATED questionnaires and the self-determination activities were completed via in-class responder technology. Data were de-identified and downloaded from the responder technology at the end of the semester. Mixed methods were used for data analysis including quantitative analyses and a qualitative approach using a phenomenological, inductive, and reflexive qualitative method whereby themes were allowed to emerge from the data. **Results:** Overall, almost 25% of the students reported never or rarely obtaining healthy sleep on average. The majority (76%) said they sometimes have healthy sleep and no students reported usually or always obtaining healthy sleep. The components of sleep health the entire sample scored highest on were timing (sleeping between 2 and 4 AM), sleep duration (between 7 and 9 h), and staying awake during the day. The areas they scored the lowest on were maintaining regular bed and wake times, spending less than 30 min awake at night, and feeling satisfied with their sleep. Qualitatively, the most frequently obtained sleep health behaviors of the intervention class were rhythmicity, prioritizing sleep, timing of sleep, and tech hygiene. The intervention class had significantly better sleep health across the entire semester and significantly better daytime alertness post-intervention. The most commonly chosen sleep health behaviors to change were sleep hygiene, tech hygiene, and stimulus control. **Conclusion:** We examined the classroom environment as a venue for promoting sleep health among college students. Given the popularity of Introduction to Psychology courses, this class is a promising avenue to deliver sleep health promotions to a large number of students. The implementation of a self-determination framework, as part of sleep health promotion, shows potential for creating a person-centered, strengths-based approach to health behavior change within this population.

## 1. Introduction

### 1.1. Sleep Disturbances in College Students

College students are at particular risk for sleep disturbances. More than 60% of college students at a large, public university were categorized as poor sleepers using the clinical cut-off of the Pittsburgh Sleep Quality Index [1]. In another sample of college students, average PSQI scores for both men (*M* = 6.38) and women (*M* = 6.69) were above the clinical cutoff of 5, suggesting that poor sleep quality is less of an exception and more normative within this group [2]. Furthermore, an estimated 9.4% of college students meet clinical criteria for insomnia [3]. Given the pervasive nature of poor sleep within this group, and the importance of healthy sleep for personal and academic development in emerging adulthood, there is a need to better understand how to promote healthy sleep in college students.

There are several reasons for poor sleep in college students. When asked to identify the reasons for their poor sleep, students most commonly list academic stress, emotional stress, and light/noise disruption [1]. Moreover, traditional-aged college students are more likely to have evening chronotypes due to age-related biological changes, preferring to rise and be active later in the day [4]. However, this circadian timing preference may not align with school and work obligations. Therefore, young adults may have to abide by an earlier schedule on workdays and follow their biological preference, a later schedule, on free days. This misalignment between biological and social time has been referred to as social jetlag and is associated with negative effects on sleep quality [4]. Lastly, students often overestimate their sleep health and downplay the importance of sleep in self-report [2,5], suggesting a lack of insight into their own sleep patterns and knowledge about the consequences of poor sleep. 

Sleep disturbance can have detrimental effects on college students. In addition to the physical, cognitive, and psychological consequences of poor sleep that affect all age groups [6,7,8], poor sleep quality can have negative effects on academic performance and social factors specific to the college environment. Over half (54%) of college students report skipping class due to poor sleep, and 46% of students report falling asleep in class on occasion [2]. College is often a period of experimentation with alcohol, and poor sleep is also associated with binge drinking and alcohol-related consequences in young adults [9].

### 1.2. Sleep Health in College Students

Though the college experience may contribute to poor sleep outcomes in college students, less is known about how to promote healthy sleep in this population. Beyond a deficit-based approach to understanding sleep, it is essential to examine sleep in this population from a positive lens in order to promote optimum sleep. *Sleep health* has been proposed as a holistic, strengths-based framing of sleep characteristics such as regularity, satisfaction with sleep, alertness, timing, efficiency, and duration that exists along a continuum [10,11]. Sleep health is not only the absence of sleep disorders or complaints, rather it encompasses all of the aspects of sleep—positive and negative [11]. Though the field of sleep research increasingly values sleep health, this framework has not often been used to examine sleep in college students. 

### 1.3. Sleep Interventions for College Students—Self-Determination Theory 

Although existing sleep interventions in college students show promise for addressing disordered sleep in this group [12,13], less is known about how to promote healthy sleep across the continuum of sleep health in college students. Self-determination theory (SDT) provides a relevant and innovative framework for designing sleep health interventions in college students. Self-determination theory is a theory of human motivation and factors that foster an individual’s innate tendencies to grow and develop [14]. An aspect of SDT that seems particularly relevant to health behavior change is that of basic psychological need satisfaction. This sub theory posits that humans have three basic psychological needs—autonomy, competence, and relatedness—and that individuals land on a spectrum ranging from satisfaction to frustration for each of these needs [15]. In order for an individual to achieve optimal growth and personal well-being, all three needs must be sufficiently satisfied [16].

Need satisfaction has been linked specifically to performance of positive health behaviors. In a qualitative study of members of a health facility, participants identified how factors that increase autonomy (e.g., self-selecting exercise activities), competence (e.g., accomplishing goals), and relatedness (e.g., having workout partners) promoted more physical activity [17]. Similarly, adults who are more satisfied in their basic psychological needs are more likely to adopt healthy strategies for eating regulation, whereas those whose needs are frustrated were more likely to engage in restrictive or uncontrolled eating [18]. With regard to sleep, basic need satisfaction has been associated with better sleep quality, less daytime dysfunction, and greater sleep duration [19]. Greater satisfaction of autonomy, competence, and relatedness appears to set the stages for enacting a variety of positive health behaviors. 

Due to the fact that basic psychological need satisfaction has been associated with greater performance of positive health behaviors and better health outcomes, interventions that promote need satisfaction should be designed. For example, in an intervention to promote motivation for physical activity within the physical education classroom, students who were exposed to a curriculum that specifically fostered autonomy, competence, and relatedness had greater success in the course than those who were exposed to the traditional curriculum [20]. Little research has focused on applying the concept of basic psychological need satisfaction to sleep health interventions, despite the known associations between need satisfaction and sleep quality [19].

The academic classroom is one venue for potentially promoting sleep health via a self-determination framework. Interventions in the classroom have targeted other health domains, such as mental health. For example, the implementation of mindfulness meditations in the undergraduate classroom have shown benefits for students with students reporting feeling calmer and less bothered by various stressors they were previously experiencing [21] as well as higher resilience and self-efficacy compared to a control group [22].

## 2. Current Study

Given that college students are at heightened risk for sleep disturbances, and that healthy sleep is associated with positive physical, cognitive, psychological, and academic benefits for this group, the goals of the current study were to describe sleep health in this group and examine the role of a class activity using self-determination theory to promote better sleep health. Within two Introduction to Psychology courses at a large public university, we used a cohort study with a mixed-methods approach to: (Aim 1) describe sleep health in this population and (Aim 2) describe and evaluate the potential of a self-determination intervention to improve sleep health in an undergraduate college sample. To our knowledge, only one pilot study has used a sleep health framework to promote better sleep in college students. Levenson and colleagues [23] aimed to increase sleep health knowledge and better psychosocial functioning through the use of didactics and sleep diaries over the course of one month [23]. They found that general knowledge about sleep health, sleep efficiency, and total sleep time improved in participants after the intervention, suggesting that sleep health is an important factor to target in college students. As a population that may be particularly prone to disrupted sleep [1], it is important to identify strengths that college students may already possess related to sleep and understand what is important for them to improve in order to feel like they are experiencing good sleep. Consequently, in the current study, we examined the classroom environment as a venue for promoting sleep health among this population using self-determination theory. The rationale for the current study was two-fold. First, by using existing infrastructure—the health psychology section of an Introduction to Psychology course—we sought to evaluate the feasibility of promoting sleep health within this modality which is in place in most Introduction to Psychology courses [24]. Given the popularity of Introduction to Psychology courses [25], this class is a promising avenue to deliver sleep health promotions to a large number of students. The second rationale for this study is that by implementing a self-determination framework as part of this intervention, we could employ a person-centered, strengths-based approach to health behavior change which is recommended for this population [26]. 

## 3. Methods

### 3.1. Participants

A cohort design was used with class activity data from undergraduate Introduction to Psychology courses. Students were undergraduates at a mid-Atlantic public university in the United States. Data were compiled post hoc from de-identified class activity responses from two classes occurring in the fall semester. One class completed a self-determination activity (intervention class) while the other did not (control class). Due to the cohort design, the classes were not randomly assigned to the activity or not. One class met at 11:00 AM twice a week while the other class met at 12:30 PM twice a week. Total sample size was *N* = 224 (intervention class [*n* = 98], and the control class [*n* = 127]). The total potential recruitment pool was 590 students (294 in the control class and 296 in the treatment class). Only students who completed all the class activities were included in the final sample. There were no inclusion or exclusion criteria other than attrition—students who completed the class activities were included in the data analysis. This study was exempt from IRB approval as it was a post hoc analysis of a naturally occurring phenomenon (a class activity) using de-identified data. Additional demographic data for participants are unavailable as the activities were completed as part of an educational exercise rather than a research study. Data were analyzed post hoc after the classes ended. 

### 3.2. Measures

#### Sleep Health

Sleep health was measured using the RU SATED scale [10]. This six-item questionnaire assesses the six key dimensions of sleep health: sleep regularity, satisfaction, alertness during the day, timing, efficiency, and duration. Items are rated on a 3-point Likert scale from 0 (*Rarely*/*Never*) to 3 (*Usually*/*Always*), with higher scores indicating greater sleep health. Evidence using a European sample of adults suggests that the scale is a valid and reliable measure for the assessment of sleep health indicators [27,28].

### 3.3. Procedure

Both the intervention and control classes completed the RU SATED sleep health questionnaire at the beginning (September) and end (November) of the fall semester. Year of the data collection is not reported to protect participant information. The intervention class also completed a self-determination activity focused on sleep health mid-semester (see below) which was delivered by the course instructor. There was a 6-week lapse between the completion of the intervention and the second RU SATED assessment. Both the RU SATED questionnaires and self-determination activities were completed via in-class responder technology where students respond to a prompt using a digital device (e.g., cell-phone, laptop computer, or iPad). The self-determination activity occurred as part of the health psychology unit. Both classes received general didactic instruction on sleep as part of the class curriculum. Data were de-identified and downloaded from the responder technology at the end of the semester.

#### Sleep and Self-Determination Intervention

A brief, 20 min, classroom intervention occurred during one class period in the semester. The intervention was designed to improve sleep experiences using tenets of self-determination theory by meeting the three basic psychological needs posited by self-determination theory: (1) competence, (2) autonomy, and (3) relatedness [13]. (1) To increase participants’ sense of competence, participants were reminded that they are the best experts regarding their own sleep experiences. Participants were prompted to identify an aspect of their sleep experience that they feel they do well with. (2) To increase participants’ sense of autonomy, participants were prompted to independently set 3 goals to improve their sleep behaviors. The SMART framework was introduced to aid students in goal setting, whereby goals should be specific, measurable, achievable, realistic, and time limited [29]. Examples of areas for sleep-behavior change were also provided, such as increasing regularity of bedtimes, obtaining 7–9 h of sleep duration, or limiting technology use before bed. To further increase autonomy, participants then independently chose which of their 3 goals they would like to adopt. (3) The final aspect of the intervention aimed to increase participants’ sense of relatedness. Participants were directed to discuss their chosen SMART goal (Specific, Measurable, Achievable, Relevant, and Timebound) with a class partner. Specifically, participants were prompted to share potential barriers to their goal and to brainstorm solutions to these barriers with their partners. A complete description of this self-determination intervention, including directions given to participants, is included in Table 1.

## 4. Data Analytic Plan

### 4.1. Quantitative Methods

Descriptive statistics were calculated for RU SATED components and total scores. Multivariate analysis of variance was conducted to examine overall group (intervention versus control) differences in RU SATED components and total scores, and a two-way repeated measures multivariate analysis of variance was conducted to examine group (intervention versus control) by time (pre- and post-intervention) interactions. For all quantitative analyses, *p* values of <0.05 were considered significant and this study was significantly powered to detect a small effect size with a power of 0.80 and alpha of 0.05. Authors performing quantitative analyses were unaware of group membership during the analyses process.

### 4.2. Qualitative Methods

The qualitative approach used a phenomenological, inductive, and reflexive qualitative method whereby themes were allowed to emerge from the data. Further, researchers engaged in reflexivity to account for the potential impacts of positionality and bias on interpretation of findings. A subsample of 98 students participated in the self-determination intervention. Students responded to 3 qualitative prompts (see above). Thematic analysis was used in the coding of qualitative data. Thematic analysis can be defined as “a method for identifying, analyzing and reporting patterns (themes) within data” [30]. Although content analysis uses a largely positivist perspective, thematic analysis uses a constructivist approach to qualitative data analysis based on the philosophy that meaning is constructed through lived experiences [31]. Further, the decision to utilize thematic analysis over content analysis was guided by the approach of these methodologies to theme construction. A content analysis approach requires that the researcher determine themes a priori, or prior to data analysis. This approach, although guided by theory, is limited in its ability to capture the phenomenological, emergent content of qualitative data. In contrast, a thematic analysis approach allows for researchers to engage in a bi-directional, emergent coding process, whereby themes emerge from the data throughout the process of analysis and, once identified, can be re-applied as necessary to attain the most representative theme–data fit outcomes. Specifically, the present study employed inductive reflexive thematic analysis [30,32] whereby the analysis was data driven, rather than guided by a predetermined theoretical framework, with attention to researcher positionality and biases relevant to coding processes and outcomes.

Clarke and Braun [33] present a 6-step process for thematic analysis, including becoming familiar with the data; generating initial codes and applying these codes to the dataset; identifying broader patterns of meaning; reviewing themes and determining if themes accurately represent the story of the data; defining and naming themes; and weaving together the narrative and data. In accordance with this process of thematic analysis, 3 raters (2 graduate students and a counseling psychology faculty member, all sleep researchers) engaged in an initial coding process whereby each coder worked on their own spreadsheet containing responses to all 3 qualitative items independently to determine which codes emerged from the data. Following this initial coding process, the 3 coders met to discuss which themes emerged and which should be retained for the next stage of the coding process. Once consensus was achieved, the 2 graduate student coders conducted a final coding of the data utilizing collaboratively constructed themes. Following this second coding process, all 3 coders met once more in order to discuss any discrepancies in coding and to make final decisions on retained themes. Two coders then engaged in final coding using the agreed upon themes and discussed any discrepancies in coding to arrive at a final qualitative dataset.

## 5. Reflexivity

Engaging in reflexive thematic analysis requires that researchers acknowledge and articulate underlying assumptions guiding their approach and interpretation of qualitative data [30]. Reflexivity asks the researcher to consider their own partiality and position in relation to the research content and process [34]. As such, the coders engaged in reflexivity during the coding process, taking note of their social locations and research biases that may influence their interpretation and assignment of themes to qualitative responses. According to Berger [35], factors such as gender identity, age, personal experiences, beliefs, and biases are all examples of domains relevant to personal reflexivity in research. Though this list is not exhaustive, it provides a template upon which researchers can begin to transfer reflexivity from solely the qualitative research domain into quantitative and mixed-methods research, such as the current study.

The three qualitative coders identify as white, cisgender and non-binary women, emerging and early middle-aged adults, with sleep research interests and backgrounds. Two coders are graduate students in a sleep research lab and one coder is a behavioral medicine researcher and health psychology course instructor. All coders have personally struggled with sleep now or in the past. Notable biases/reflections that arose for the researchers during the coding process were expectations of undergraduate students’ knowledge and application of healthy sleep behaviors and overall commitment to wellness. The coders maintained an awareness of their biases as sleep researchers throughout the coding process, giving their best effort toward assigning themes which emerged from the data rather than from their own biases informed by past research and beliefs about sleep.

## 6. Results

### 6.1. Aim 1: Describe Sleep Health in an Introductory Psychology Undergraduate College Sample

#### 6.1.1. Quantitative Results

**Figure 1:**
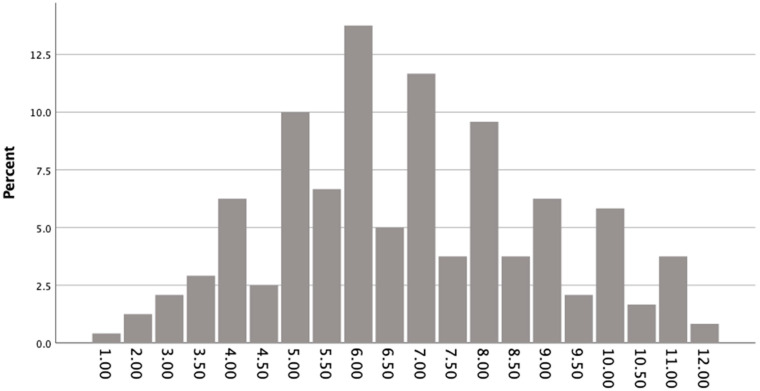
Total Sample’s Mean RU SATED Total Scores Ranging from 0 to 12 (*N* = 224).

**Figure 2:**
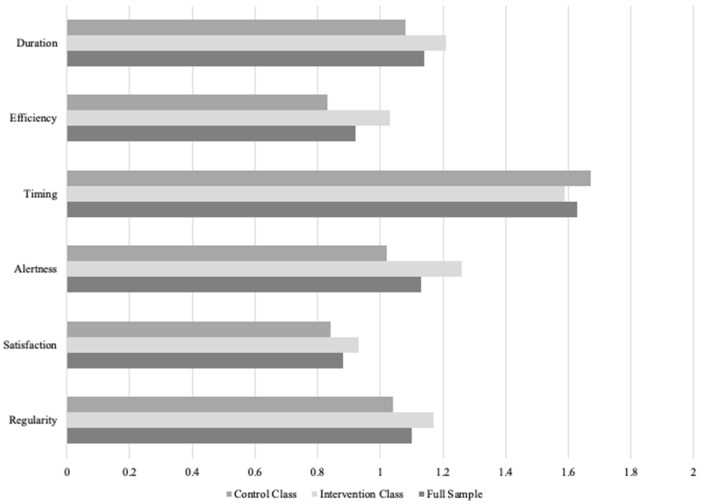
RU SATED Component Scores Averaged across the Semester for the Total Class (*N* = 224), the Intervention Class (*N* = 97), and the Control Class (*N* = 127).

**Total Sample.** The mean RU SATED sleep health score for the total sample averaged across the whole semester (across the two time points) was *M* = 6.82 (*SD* = 2.13) out of a total possible range from 0 to 12. On average, 24.20% of participants reported rarely or never obtaining healthy sleep, 75.80% reported sometimes obtaining healthy sleep, and 0% reported usually or always obtaining healthy sleep (see Figure 1). Among the six components of healthy sleep measured by RU SATED, timing was rated the highest across the semester (asleep between 2 and 4 AM) and satisfaction was rated the lowest (see Figure 2) There was no significant difference in sleep health for the full sample from the start of the semester to the end of the semester, *t*(150) = 0.00, *p* = 1.00.

**Intervention Class.** The mean RU SATED sleep health score for the intervention class averaged across the whole semester was *M* = 7.26 (*SD* = 2.03) out of a total possible range from 0 to 12. On average, 17.90% of participants reported rarely or never obtaining healthy sleep, 82.10% reported sometimes obtaining healthy sleep, and 0% reported usually or always obtaining healthy sleep. Among the six components of healthy sleep measured by RU SATED, timing was rated the highest across the semester (asleep between 2 and 4 AM) and satisfaction was rated the lowest (see Figure 2)

**Control Class.** The mean RU SATED sleep health score for the control class averaged across the whole semester was *M* = 6.45 (*SD* = 2.15) out of a total possible range from 0 to 12. On average, 29.50% of participants reported rarely or never obtaining healthy sleep, 70.50% reported sometimes obtaining healthy sleep, and 0% reported usually or always obtaining healthy sleep. Among the six components of healthy sleep measured by RU SATED, timing was rated the highest across the semester (asleep between 2 and 4 AM) and efficiency was rated the lowest (spending less than 30 min awake at night; see Figure 2).

#### 6.1.2. Qualitative Results

Themes which emerged in response to the question “What is something you already do well regarding your sleep habits?” include: general sleep timing; sleep hygiene; bedtime routine; daytime activities; rhythmicity; tech hygiene; prioritizing sleep duration; stimulus control; creating an environment conducive to sleep; and other (e.g., monitoring sleep, substance use). Of these themes, the most commonly endorsed were rhythmicity (24.29%), prioritizing sleep duration (22.14%), general sleep timing (11.43%), and tech hygiene (10.71%). The theme “rhythmicity” captured those responses reflective of regularity and consistency of wake and sleep timing. Common responses associated with this theme fell into categories including waking/sleeping at the same exact times daily, setting a consistent window of time during which to fall asleep each night, and specifically utilizing an alarm to wake at the same time each morning. Further, the theme “prioritizing sleep duration” includes responses directed toward acquiring a certain amount of sleep, such as sleeping between 6 and 8 h per night, limiting time engaged in schoolwork at night to allow time for sleep, and getting “enough” sleep. Notably, the sleep duration times mentioned did not necessarily fit within recommended guidelines of 7 to 9 h per night. “General sleep timing” represents responses geared toward tangible sleep times including setting a specific bedtime and planning a bedtime based upon the time one needs to wake up the next morning. Lastly, the theme “tech hygiene” refers to efforts given toward reducing technology use in bed. Responses coded as “tech hygiene” within the current study included categories such as minimizing blue light exposure, utilizing nighttime-specific phone features to discourage phone use in bed, and placing the phone out of reach when getting into bed. See Table 2 for themes, representative responses, and percentage of the sample endorsing responses associated with themes.

#### 6.1.3. Integration of Quantitative and Qualitative Results

There were similarities and differences between the qualitative and quantitative findings for the intervention class. When identifying what they are doing well, the most frequently endorsed behaviors of the intervention class in descending frequency were rhythmicity, prioritizing sleep, timing of sleep, and tech hygiene. According to the quantitative results, they most frequently endorsed sleep timing, staying awake during the day, sleep duration, and regularity of sleep and wake times.

### 6.2. Aim 2: Describe and Evaluate the Effectiveness of a Self-Determination Intervention to Improve Sleep Health an Undergraduate College Sample

#### 6.2.1. Quantitative Results

Independent samples *t*-tests revealed that the intervention class had significantly better sleep health averaged across the entire semester compared to the control class, *t*(205) = 2.76, *p* = 0.006. Additionally, the intervention class had significantly more regular sleep (going to bed and getting out of bed within an hour each day) compared to the control class at the start of the semester, *t*(200) = 2.18, *p* = 0.03. Post-intervention, the intervention class reported significantly better daytime alertness (staying awake all day without dozing) compared to the control class, *t*(161) = 2.64, *p* = 0.009. No significant differences were found between the two groups in terms of the other RU SATED component variables or RU SATED summed scores either pre-, post-, or change across the semester, *p* > 0.05.

#### 6.2.2. Qualitative Results

When students were asked to choose three areas in which they might want to make SMART goals to improve their sleep, several themes emerged: monitoring sleep, tech hygiene, sleep hygiene, daytime activities, strategic napping, stimulus control, bedtime routine, sleep duration, consistent sleep schedule, and other (e.g., earlier bedtime, earlier wake time, substance use). Many of these are also reflected in the themes that emerged when students were asked what they already do well regarding their sleep habits. The most common areas that students wanted to work on were sleep hygiene (20.92%), consistent sleep schedule (17.57%), tech hygiene (17.15%), and stimulus control (15.48%). “Sleep hygiene” includes goals that reflect commitment to improving conditions that affect sleep, such as reducing naps, caffeine and nicotine intake, avoiding the act of watching the clock when trying to sleep, and limiting exposure to blue light and stimulating activities before bed. Responses in this theme typically reflected commitment to not drinking or smoking right before bed, eating healthy foods, and eliminating naps from students’ daily routines. The theme “consistent sleep schedule” refers to goals of making the timing of wake and sleep more fixed and stable across time. Typical responses fell into the categories of going to bed at the same time every night, waking up at a certain time, or improving both sleep and wake times simultaneously. Tech hygiene, as defined above, was displayed in statements such as keeping phones out of the bedroom, not using phones before actively trying to sleep, turning off televisions and other electronics before bed, and replacing those activities with more relaxing ones (e.g., reading). The theme of “stimulus control” refers to responses that prioritized only using the bed for sleeping and getting out of bed if one does not feel sleepy. To see representative responses and response percentages for each theme that emerged, see Table 3.

After identifying three areas that students would like to work on regarding their sleep habits, they were asked to select just one of the goals that they listed to write a SMART goal for. The themes that emerged for this question were: stimulus control, tech hygiene, sleep timing, daytime routine, sleep hygiene, regular sleep schedule, bedtime routine, prioritizing sleep duration, and other (e.g., wake time adjustment, finding new spaces for daily tasks). The most commonly chosen priorities for students included sleep hygiene (22.22%), tech hygiene (19.44%), and stimulus control (14.81%). These themes are defined above. For this question, the theme of sleep hygiene included responses that reflected a desire to engage in health daytime habits that might help sleep (e.g., exercising before bed, eating healthily). The theme of “tech hygiene” included similar responses to Question 2: putting phones away before bed, limiting television time before and during sleep, and leaving electronics farther away from the bed to charge overnight. Finally, “stimulus control” for this question included responses that indicated priorities of getting out of bed when unable to fall asleep and using the bed only for sleep. Table 4 details percentages of responses for each theme as well as representative responses.

#### 6.2.3. Integration of Quantitative and Qualitative Results

Efficiency (spending less than 30 min awake at night) and satisfaction with sleep were the areas that were rated lowest quantitatively for the intervention class. Although wakefulness during the night and overall sleep quality/satisfaction were not identified in the top three SMART goals, or the selected sleep goal, the top qualitative themes could be seen as behaviors that could lead to decreased wakefulness and better quality/sleep satisfaction. In particular, sleep hygiene, a consistent sleep schedule, tech hygiene, stimulus control, and sleep timing could be seen as behaviors that could reduce wakefulness and promote less disrupted sleep.

## 7. Discussion

### 7.1. Overview of Results

The first aim of this study was to describe the sleep health of an undergraduate sample. Overall, almost 25% of the students reported never or rarely obtaining healthy sleep on average. The majority (76%) said that they sometimes have healthy sleep and no students reported usually or always obtaining healthy sleep. For the entire sample, the components of sleep health students scored the highest on were timing (sleeping between 2 and 4 AM), sleep duration (between 7 and 9 h), and staying awake during the day. The areas students scored the lowest were maintaining regular bed and wake times, spending less than 30 min awake at night, and feeling satisfied with their sleep. Qualitatively, the most frequently obtained sleep health behaviors of the intervention class in descending frequency were rhythmicity, prioritizing sleep, timing of sleep, and tech hygiene.

The second aim of this study was to describe and evaluate a self-determination intervention to promote sleep health among undergraduate students. Some improvements in sleep health were seen in the intervention class compared to the control class. Specifically, the intervention class had significantly better sleep health across the entire semester (although they also started with better sleep health) and significantly better daytime alertness post-intervention. Qualitative data from the intervention revealed that students identified a variety of areas of their sleep health that they could change (10 broad themes). The most commonly chosen sleep health behaviors to change were sleep hygiene, tech hygiene, and stimulus control.

The current study adds new information to the understanding of overall sleep health of college students. Prior research has primarily focused on estimating disturbed or disordered sleep [36,37]. The limitation of a deficit-based approach to sleep in this population is missed information about healthy sleep in this group as the absence of disordered sleep does not equate with healthy sleep. Consequently, in the current study, we had the opportunity to describe sleep along a continuum from poor to good, across multiple components. Despite the opportunity to identify healthy sleepers in this college student sample, no students reported usually or always obtaining healthy sleep. Although there are currently no other estimates of total sleep health in undergraduate populations, the current sample’s reports of poor sleep health are qualitatively similar to existing research on sleep in undergraduate populations. For example, 25% of students reported never or rarely obtaining healthy sleep. Similarly, research suggests most college students have poor sleep quality [1,2] and 9.4% even meet criteria for insomnia [3].

There is also little research describing sleep-related behaviors in college students, such as sleep timing, sleep duration, and sleep hygiene behaviors. Notably, the current study corroborates other research which suggests college students have difficulty regulating their bed and wake times [1]. Out of numerous aspects of sleep health, one of the behaviors that the current sample of college students struggled with the most quantitatively and qualitatively was maintaining regular bed and wake times. Similarly, undergraduate populations are vulnerable to social jet lag whereby they have much later wake times on weekends [38]. It seems that sleep regularity is particularly difficult, yet important, for college students, likely due to both biological (chronotypes) and social (school schedule and social demands) factors. Additionally, students in the current sample noted a desire to improve their tech hygiene as a means to improving sleep. As using smartphones in bed allows light to disrupt sleep and the content to create sleep-disrupting cognitive arousal [39], it is sensible that college students’ technology use is a significant factor in their sleep. The importance of tech hygiene is increasingly important to college populations given the transition to digital learning mediums, and, unfortunately, is under-addressed in the current literature.

The current study’s finding that a psychological intervention shows potential for improving sleep health provides new information about the importance of (1) promoting sleep health within this group and (2) enhancing health interventions with psychological motivation theories. Previously the efficacy of interventions for disordered sleep within this group have been established [12,13]. Furthermore, existing research has shown that solely delivering information about sleep is less effective (i.e., sleep hygiene techniques were less effective in improving various aspects of sleep [12]). Therefore, the current study illustrates a potential intervention that includes psychological techniques, such as cognitive, behavioral, or self-determination techniques. The integration of self-determination theory into the current intervention is novel and warranted. Meeting the basic psychological needs posited in self-determination theory has already been associated with better sleep in college students [19], and self-determination interventions have already been applied successfully to improve other health behaviors [20]. Because broad interventions for college student sleep are necessary, the current study’s self-determination intervention within an already existing infrastructure—an Introduction to Psychology Course—is a progression of current sleep intervention research with an eye towards dissemination.

### 7.2. Theoretical and Clinical Implications

Sleep health is critically important and underrecognized as a correlate in overall health and a framework to promote optimum sleep. Given the heightened risk college students face for poor sleep, it is critical and necessary to transition from focusing on sleep disorders to the position of sleep health, which provides a more holistic, health-oriented perspective on sleep. The current study provides information on one approach in which sleep health can be targeted and promoted as part of the Introduction to Psychology curriculum. Importantly, it affords the opportunity for researchers and clinicians to be able to intervene at multiple levels of sleep (i.e., regularity; duration). Further, the present study highlights the multidimensionality of sleep through a positive, strengths-based approach. Sleep as a multicomponent health behavior means that individuals will inevitably possess both strengths and weaknesses in various aspects. Students, for example, may exhibit healthy sleep behaviors in one domain and not another. This framework allows students to harness and enhance their strengths while also providing space and resources to promote and address other domains of sleep that could be improved. In addition, the present study was the first to combine self-determination theory with a health behavior intervention in a classroom activity pertaining to sleep health. Our findings highlight the importance of addressing autonomy and social support, as well as enhancing motivation, for this group, as a means to promote healthy sleep.

### 7.3. Limitations and Future Directions

Study findings should be interpreted in the context of several limitations. First, the use of a cohort design precludes any conclusion about causality due to the inability to control for potential confounds and randomly assign participants to groups. Although this design provided information about students’ perception and experience of sleep health within their classroom setting, future research using a randomized, controlled design is needed. By not randomizing, we could not control for biases such as better initial sleep health in the intervention class. The inclusion of participants from two discrete class periods introduces potential selection bias in the recruitment of the overall sample. Limiting participant inclusion to students who elect to take a course later in the morning/early in the afternoon precludes the recruitment of a more representative range of college student sleep schedules based on activity patterns. Furthermore, the study sample may be biased towards students who attend class as only those who completed the in-class activity were included in the data analysis. Future researchers should space out the timings of eligible course offerings and include all class students if possible as well as non-psychology students. A second limitation of this study pertains to the expertise of the course instructors regarding sleep health. One of the included psychology courses was taught by a professor with a behavioral sleep medicine background, this could have unintentionally led to a more comprehensive and higher quality sleep health education for the affected participants regardless of grouping for the intervention. This limitation could have been addressed through the integration of a structured lesson plan for any class that involved discussion of sleep health principles. Lastly, this study did not seek to obtain more detailed information regarding participants’ sleep patterns through a sleep diary or otherwise. This information could have helped corroborate claims endorsed by students through the RU SATED measure and allowed for more accurate comparison between interventions and control groups. Given the post hoc analysis of an already existing class activity, there were obvious research design limitations. In general, future research should continue to incorporate and promote sleep health in class curriculums and explore different ways in which it can be taught in the classroom.

## 8. Conclusions

The current study addressed a critical need, promoting healthy sleep, among college students using an already existing infrastructure—the undergraduate classroom. Despite limitations arising from the post hoc analysis of existing class data, the mixed-methods results provide important information about sleep health in this group. Specifically, the results suggest that this sample of undergraduate students is not obtaining healthy sleep. However, students identified areas of strength, as well as goals for improving their sleep. The integration of a self-determination framework for increasing students’ autonomy, competence, and relatedness shows promise for promoting sleep health within this group.

## Figures and Tables

**Table 1:** Outline of Self-Determination Intervention.

Psychological Needs in Self-Determination Theory	Intervention
Competence	“There are probably a lot of things you’re already doing well regarding your sleep habits. If you think about it, you have come this far because you’re doing something right! Your personal strengths and knowledge about yourself as an individual will make you successful in achieving goals related to improving your sleep habits. What is something you already do well regarding your sleep habits?”
Autonomy	Review SMART goal setting
	“Brainstorm three areas where you might want to make SMART goals to improve your sleep. You could focus on an area of sleep that you are already doing well in or an area of sleep that you are struggling. Some examples of areas to work on could be: regular bed/wake times, getting 7-9 h of sleep, limiting technology in bed, etc.”
	“You are the most knowledgeable about what you do well, what you need, and your specific context. Out of the three areas you listed, choose which area you would like to focus on changing. Write a SMART goal for this chosen area.”
Relatedness	“Discuss with a class partner which SMART goal you chose. What do you anticipate could make completing that goal difficult? Brainstorm solutions to possible barriers with a partner.”

**Table 2:** Question 1 qualitative themes, representative responses, and percentage of sample endorsement.

Question 1: What is Something You Already Do Well Regarding Your Sleep Habits?		
Theme	Representative Response	% of Sample
Rhythmicity/Timing of Wake and Sleep	“Something that I already do well regarding sleep habits is that I sleep and wake up around the same time everyday.”	24.21%
Prioritizing Sleep Duration	“I get the suggested amount of sleep for my age every night.”	22.14%
General Sleep Timing	“I try to go to sleep no later than 1am, that’s my stopping point.”	11.43%
Tech Hygiene	“I don’t look at my phone before going to sleep as the blue light emitted from a cell phone can make it hard to go to sleep and keep me up longer.”	10.71%
Sleep Hygiene	“I don’t drink caffeine towards the end of the day.”	7.86%
Bedtime Routine	“I have a nightly routine that helps prepare my body and mind for rest.”	7.14%
Other (monitoring, substances)	“Take melatonin to fall asleep faster/easier.”	7.14%
Daytime Activities	“I always try to do my homework early so that I am not up late at night working on it.”	3.57%
Stimulus Control	“Something I already do well regarding my sleep habits is sleep when sleepy. When I get really tired I just head to bed and shlump.”	2.86%
Creating an Environment Conducive to Sleep	“I sleep with two fans on and have many pillows and blankets in my comfy bed.”	2.86%

**Table 3:** Question 2 qualitative themes, representative responses, and percentage of sample endorsement.

Question 2: Brainstorm Three Areas That You Might Want to Make SMART Goals to Improve Your Sleep.		
Theme	Representative Response	% of Sample
Sleep Hygiene	“quit smoking right before bed. no naps, makes me less tired when bedtime actually hits.”	20.92%
Consistent Sleep Schedule	“I would like to get 7-9 h of sleep on the daily to feel fully awake and aware in the morning.”	17.57%
Tech Hygiene	“The last goal that I would like to make would be to limit my technology usage before bed. When I lay down to go to bed, I normally stay awake for another 30ish minutes going through my phone which I know is very bad for sleep hygiene, so I would like to fix this.”	17.15%
Stimulus Control	“sleeping when sleepy, I have this habit of fighting my sleep during the day when I know I need some shut-eye.”	15.48%
Sleep Duration	“The first area I would like to work on is the amount of time I sleep. I would like to get at least 8 h a night but that’s asking a lot”	8.37%
Daytime Activities	“Lastly I want to work on keeping my daytime routine the same since being in college everyday is not the same so I would like to work on that.”	8.37%
Other (substances, earlier wake/sleep time)	“I also need to avoid alcohol as I’m unable to sleep at all without it and that’s super unhealthy!”	5.86%
Bedtime Routine	“I also want to come up with a sleep ritual for before I go to bed, maybe it could improve the quality of my sleep.”	3.35%
Monitoring	“Keeping a sleep diary.”	1.67%
Strategic Napping	“I take a lot of naps during the day, I could cut back on those for better sleep at night.”	1.26%

**Table 4:** Question 3 qualitative themes, representative responses, and percentage of sample endorsement.

Question 3: Out of the Three Areas You Listed, Choose Which Area You Would Like to Focus On Changing? Write a SMART Goal for This Chosen Area.		
Theme	Representative Response	% of Sample
Sleep Hygiene	“I will not nap in between my classes on Wednesdays (my busiest days) for the next two weeks and see how this impacts my sleeping habits/productivity”	22.22%
Tech Hygiene	“I will put my phone and computer away 15 min before bed at night. Instead of using social media and technology before bed I will pick up reading as a better way to clear my mind at night.”	19.44%
Stimulus Control	“I will do my homework only at my desk and leave my bed for relaxation and sleep purposes only for the next two weeks so that I can test if I am able to get better sleep as a result.”	14.81%
Sleep Timing	“I will sleep between 10:30-12 on weekdays and before 2 on weekends for the rest of the semester.”	12.04%
Regular Sleep Schedule	“I will go to bed and wake up at the same time for the next 2 weeks.”	11.11%
Daytime Routine	“I will work out every day to help me feel more tired.”	8.33%
Other (wake time adjustment, finding new spaces for daily tasks)	“Mainly I would like to fix the amount of time I spend in my bedroom. I don’t think that it is conducive to getting work done and it makes it harder to get to sleep when I need to be sleeping. My goal would be to change this behavior by establishing other locations that I can get my homework done and if I want to spend time watching television and relaxing during the day I should change that space as well.”	5.56%
Bedtime Routine	“I will practice relaxing stretches 15 min before going to bed each night for the next 4 weeks.”	3.70%
Prioritizing Sleep Duration	“I will strive to get at least 8 h of sleep.”	2.78%

## Data Availability

Not applicable.
